# Instagram and Seizure: Knowledge, Access, and Perception of Circulating Information on the Internet

**DOI:** 10.7759/cureus.41664

**Published:** 2023-07-10

**Authors:** Helen A. O Popoola-Samuel, Hamsa Priya Bhuchakra, Tamara Tango, Naisargi Dharmendrakumar Pandya, Kiran Lakshmi Narayan

**Affiliations:** 1 Neurology, Rush University College of Health Sciences, Chicago, USA; 2 Neurology, Apollo Institute of Medical Sciences and Research, Hyderabad, IND; 3 Neurosurgery, Faculty of Medicine, Universitas Indonesia, Jakarta, IDN; 4 Neurology, Faculty of Medicine, Universitas Indonesia, Jakarta, IDN; 5 Internal Medicine, Faculty of Medicine, Universitas Indonesia, Jakarta, IDN; 6 Neurology, Dr. Kiran C. Patel Medical College and Research Institute, Bharuch, IND; 7 Neurology, Aasare Hospital, Nelamangala, IND

**Keywords:** seizure, circulating information, internet, perception, access, knowledge, instagram, epilepsy

## Abstract

Introduction:Social media has many advantages as a tool in the healthcare industry. On the other hand, the disadvantages of using social media to obtain data include the unequal quality of uncontrolled and unchecked content. Our study aimed to assess the accuracy of the information on epilepsy or issues relevant to epilepsy observed on social media.

Methods:A semi-structured online questionnaire was employed, which incorporated a reliability index and a global quality index. Five different hashtags were used to obtain the Instagram posts, i.e., #seizure, #seizures, #seizuredisorder, #seizureawareness, and #seizurefree.

Results: A total of 431 Instagram posts related to seizures were collected, of which 76.8% contained true information. Moreover, 6.3% of the total posts (n = 27) contained promotional content. The data were then divided into groups A and B based on their involvement in active patient care. Statistically, group A posts had more correct information being circulated when compared to group B (p = 0.000387). Group A posts also had a statistically significant higher mean global quality score (p = 0.0033).

Discussion:This current study provides a comprehensive reference on the usage of social media in epilepsy to assess the veracity of the information on epilepsy and related topics.

## Introduction

Seizures are transient disruptions of neurological functioning caused by the aberrant firing of neurons. This symptom is often encountered in clinical practice. In the United States, at least 170,000 individuals experience their first seizure annually [1]. Moreover, around 10% of the overall population encounters at least one seizure during their lifetime, with the most frequent incidence occurring during early childhood and late adulthood. Adult-onset seizures can be caused by several etiologies, such as trauma, infections of the central nervous system, space-occupying lesions, strokes, metabolic diseases, and medications. However, seizures that start in childhood are usually idiopathic. Additionally, the etiology and clinical picture of seizures in adults involve pharmacological considerations regarding the initiation and cessation of treatment that are distinct from those for younger patients [2].

The term epilepsy is typically used to describe recurrent unprovoked seizures [1]. According to The International League Against Epilepsy (ILAE), epilepsy is defined as a brain disease characterized by having at least two unprovoked seizures with a time interval of more than 24 hours, or experiencing one unprovoked seizure along with a likelihood of subsequent seizure comparable to general recurrence risk (at least 60%) after two unprovoked seizures occurring within the next 10 years, or having a diagnosis of epilepsy syndrome [3]. Epilepsy affects around 50 million people globally. The incidence rate of epilepsy was 61.4 per 100,000 person-years, with an incidence in low- and middle-income countries (LMIC) and high-income countries (HIC) at 139.0 and 48.9, respectively [4]. Epilepsy can be classified based on seizure type (focal onset, generalized onset, and unknown onset) and seizure type (focal, generalized, combined generalized and focal, as well as unknown) [5].

Patients with epilepsy often experience stigma, such as embarrassment about having epilepsy, discrimination by relatives, difficulty to find a job, restrictions at school, and social isolation. Consequently, it may discourage them to seek treatment and affect their quality of life. Several factors contribute to this stigma, including a low level of information regarding epilepsy, a low educational level, a low socioeconomic status, religious reasons, and living in a rural area [4]. 

Social media now plays a crucial role in the lives of an ever-growing number of individuals globally. Twitter reported having over 313 million active users in 2016. In addition, based on a survey conducted between 7 March 2018 and 10 April 2018 among US teens, YouTube and Instagram were used by 85% and 72% of US teens, respectively [6]. 

Healthcare professionals are becoming more interested and involved in the use of social media. Healthcare professionals may use social media to promote health awareness, educate patients, develop professional networks, and communicate with relatives [7]. Social media’s merits as a platform to share health-related information allow anyone to gather information from any user, including non-healthcare professionals. This may contribute to misconception about health information as the information is unregulated and unchecked [6,8]. A previous study conducted by Meng et al. analyzed 403 epilepsy-related posts on Facebook and Twitter, and the result showed that 48% of the posts focused on epilepsy treatment as well as the correction of epilepsy-related misconceptions [8]. In addition, a study conducted by Jiang et al. analyzed 109 epilepsy-related videos in TikTok and found that 26 out of 47 event videos misinterpreted non-epileptic events as epileptic events [9]. A study that analyzed epilepsy-related posts on other social media platforms, such as Instagram, has not yet been explored.

Therefore, we aimed to evaluate the relevance of information related to seizure disorders available on Instagram by using top hashtags and assess the quality and reliability of these posts to pinpoint any gaps in patient-physician communication and public knowledge of certain issues and treatment options.

## Materials and methods

To identify the gaps and areas for improvement, a web survey was conducted to assess the accuracy of the information on epilepsy or issues relevant to epilepsy observed on social media. The data collection period lasted for 16 days (from December 5th to December 20th, 2022). For this web-based study design, human participants were not involved. Instead, hashtags on public Instagram accounts were analyzed to see the reliability of the information linked to the hashtags. The first step of this process was to collect data via Google Forms, which was automatically assembled into a Microsoft Excel sheet (Microsoft Corp., New York, USA). Next, each researcher was allotted one of the following hashtags: #seizure, #seizures, #seizureawareness, #seizurefree, and #seizuredisorder. After that, five researchers went to Instagram on their cellular devices, searched the seizure-related content using the allotted hashtags, and answered the survey questions on a Google Form for each Instagram post and/or reel. A total of ten Instagram posts and/or reels per day per researcher, which contains the top five posts and/or reels and five recent posts and/or reels daily. Throughout the study period, 100 posts and/or reels per researcher were collected, and the Excel sheet was used to collect responses to the posts reviewed. 

The following survey questions were used:

Q1. Is the post relevant to the hashtag?

Q2. Was the content a post or video/reel?

Q3. What is the duration since posted?

Q4. What is the absolute number of likes?

Q5. What is the absolute number of comments?

Q6. What is the number of followers with the absolute number?

Q7. Who posted the content (e.g., physician, parent, dietician, and more)?

Q8. Was the description of the disease or surgery posted by a physician?

Q9. Does the post describe or discuss information on symptoms, prevalence, cause/etiology, diagnosis, or screening? 

Q10. Does the post describe or discuss information on prevention, treatment, mortality, rehabilitation, or support groups?

Q11. Is there information about the people/patients sharing their own experiences?

Q12. Is there information about the parent sharing their family experience?

Q13. Is the information true or false or cannot be determined (referring to the WHO, CDC, and ILAE definitions of seizure and/or epilepsy)?

Q14. Is it a digitally created image?

Q15. Does the post have promotional content by a pharmaceutical company or by physicians?

Q16. Give a Global Quality Score for each Instagram post. The score of Global Quality Score depends on the quality, flow, content of information, and usefulness of the information. The Global Quality Score ranges from 1 to 5. The score given to each Instagram post should match the description of the respected score in the Global Quality Score (Table 1).

**Table 1:** Global quality score. Source: [10].

Score	Description of global quality score
1	The content is of poor quality and poor flow, most information is missing, and the content is not useful for the patient at all.
2	Generally, the content is of poor quality and poor flow. Some information is listed, but much information is missing. It provides limited information for the patient.
3	The content is of moderate quality with a suboptimal flow. Some information is adequately discussed, but the rest is poorly discussed. It provides somewhat useful information for patients.
4	The content is good in quality and flow. Most information is listed, with some information not covered. The content is useful for patients.
5	The content is excellent in quality and flow. It provides very useful information for patients.

Q17. Give a reliability score for each Instagram post. For every yes answer to every question on the reliability score (Table 2), the Instagram post obtains one point. The reliability score ranges from 1 to 5.

Q18. Is there any other information needed?

**Table 2:** Reliability score. Source: [11].

Reliability score
Are the aims of the content clear?
Is it clear regarding the sources of the information (other than the author)?
Is the information presented balanced and unbiased?
Are there any additional sources of information listed for patient reference?
Does the post refer to areas of uncertainty?

Regarding guidelines, the World Health Organization (WHO) [12], Centers for Disease Control and Prevention (CDC) [13], and ILAE [4] were used as references for source information when related. When conducting this study, a proforma was made, and all authors analyzed an equal number of Instagram posts. Our inclusion criteria were posts with the above hashtags (#seizure, #seizures, #seizureawareness, #seizurefree, and #seizuredisorder) and posts written in English. We excluded posts that did not contain data relevant to seizures. The engagement to the seizure-related posts was estimated based on the number of likes and comments.

Then the data were divided into groups A and B based on their involvement in active patient care. Group A consisted of the posts posted by doctors, the health and wellness industry, nurses, neuroscience physiotherapists, and dieticians. While group B consisted of posts created by survivors or people suffering from the disease, pharmaceutical companies, organizations, communities, foundations, advocates, public figures, athletes, personal accounts, seizure scholarship recipients, book agencies, chiropractic, and epilepsy wellness coaches.

Any disagreements between the authors were resolved via a mutual discussion or by consulting the senior author. No ethics committee approval was needed for the study as there were no direct human/animal subjects involved. 

Data from the survey was then transferred into Excel and analyzed using IBM® SPSS® Statistics version 23.0 (IBM Corp., New York, USA). The statistical analysis used in this study was the unpaired t-test. When assessing the association between group types (group A and group B) and the global quality score, group types acted as independent variables while the global quality score acted as dependent variables. When assessing the association between group types (group A and group B) and reliability score, group types acted as independent variables while reliability score acted as dependent variables. A p-value <0.05 was considered as statistically significant.

## Results

A total of 431 Instagram posts related to seizure disorder were collected and analyzed by the authors. The number of posts analyzed per hashtag has been summarized in Table 3. 

**Table 3:** Number of relevant posts under each hashtag.

Hashtag name	Post-analyzed	Relevant posts
#seizure	70	70
#seizures	99	99
#seizuredisorder	101	101
#seizureawareness	71	71
#seizurefree	90	90
Total	431	431

The posts were divided into read-only posts and videos/reels. The majority were in the form of a post (86.54%), and the proportion of digitally created images and non-digitally created images was similar. From the 431 posts included in our study, there were a total of 505,368 likes and 420 comments. The majority of the posts were made by the health and wellness industry (35.0%), followed by survivors (32.7%), organizations, communities, foundations, and advocates (15.1%), and doctors (10.2%). This is illustrated in Table 4.

**Table 4:** Characteristics of the Instagram posts analyzed (n = 431).

	Number of posts	% of Total
Type of posts		
Image/post	373	86.5
Videos/reels	58	13.5
Digitally created image		
Yes	213	49.4
No	218	50.6
Number of posts based on its likes		
<50 Likes	257	59.6
50–100 Likes	86	20.0
100–500 Likes	50	11.6
>500	38	8.8
Number of posts based on its comments		
<50 Comments	413	95.8
50–100 Comments	7	1.6
100–500 Comments	8	1.9
>500 Comments	3	0.7
Posted by		
Doctor	44	10.2
Health and wellness industry (including hospital and hospital staff)	151	35.0
Nurse	1	0.2
Neuroscience physiotherapy	1	0.2
Dietician	3	0.7
Survivors/persons suffering from the disease	141	32.7
Pharmaceutical company	0	0.00
Organization, community, foundation, advocate	65	15.1
Others (public figure, athlete, personal account, seizure scholarship recipient, book agency, chiropractic, epilepsy wellness coach)	25	5.8

On analyzing the content of the posts, we found that most posts were about patient experiences (39.2%), followed by symptomatology (37.8%), and then treatment (20.0%) (Table 5).

**Table 5:** Type of content being circulated about the seizure.

Type of content	Number of posts	% of Total
Explanation by a doctor	42	9.7
Etiology	66	15.3
Prevalence	45	10.4
Symptoms	163	37.8
Diagnosis	68	15.8
Screening	15	3.5
Prevention	34	7.9
Treatment	86	20.0
Mortality	21	4.9
Rehabilitation	13	3.0
Support groups	41	9.5
Patient who shared about his or her experience	169	39.2
Parents sharing their experience with a family member	64	14.9

**Figure 1:**
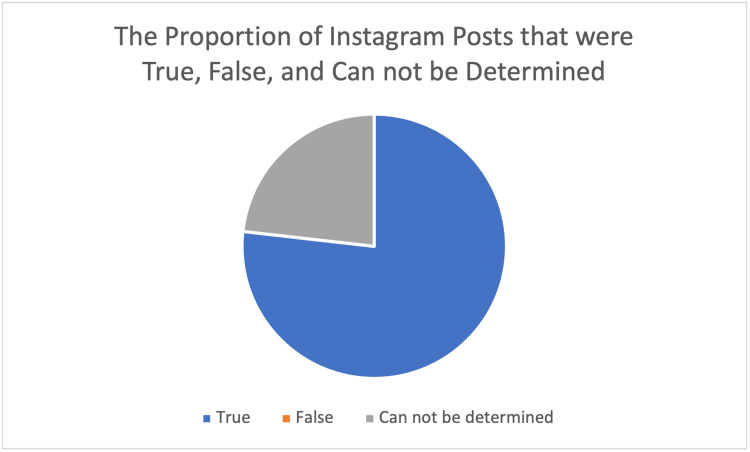
The proportion of Instagram posts that were true, false, and cannot be determined.

Figure 1 depicts the number of posts that were true, false, and cannot be determined. The information is true if the seizure/epilepsy-related post is in accordance with WHO, CDC, and ILAE’s definition of seizure/epilepsy. The information in the Instagram post is false if it contains information that is against the WHO, CDC, and ILAE’s definition of seizure/epilepsy. The information in the Instagram post cannot be determined if there is no information related to WHO, CDC, and ILAE’s definition of seizure/epilepsy. Most of the posts were true (76.8%). We did not find any false information among Instagram posts. Moreover, as Instagram is a platform to promote content, we also assessed the proportion of promotional content. Only 6.3% of the total posts (n = 27) contained promotional content.

Each author then independently analyzed the global quality score (Table 6) and reliability score of the total Instagram posts (Table 7).

**Table 6:** Global quality score.

		Number of posts	%
1	Poor quality and flow, much missing information, and not useful for the patients	131	30.4
2	In general, the posts are poor quality and flow. Some information is listed with many missing important topics. Provide limited use to the patient.	106	24.6
3	Moderate quality with suboptimal flow. Provide adequate information. Others are poorly discussed. Somewhat useful for the patient.	146	33.9
4	Good quality and good flow. Provide the majority of relevant information, but not all topics being covered. Provide useful information for the patient.	45	10.4
5	Excellent quality and flow. Provide very useful information for the patient.	3	0.7

**Table 7:** Reliability score.

		Number of posts	%
1	Clear aims	170	39.4
2	Use reliable sources of information (other than the author)	93	21.6
3	Information is presented balanced and without bias	122	28.3
4	Provide additional sources of support and information	44	10.2
5	Refer to areas of uncertainty	2	0.5

A comparison of correct information being circulated between group A and group B is illustrated in Table 8. Statistically, there was a significant difference in correct information being circulated between groups A and B (p = 0.000387).

**Table 8:** Comparison of correct information being circulated between groups A and B.

	Group A (n = 200)	Group B (n = 231)
No of correct posts	168	163
Percentage	84.0%	70.3%
p value = 0.000387		
Z score = −3.4		

Moreover, a statistical analysis using an unpaired t-test was conducted to compare the quality and reliability of the Instagram posts being circulated among groups A and B. There was a statistically significant difference in the mean global quality score in groups A and B (p = 0.0033), with a mean difference of 0.29 and a 95% confidence interval ranging from 0.10 to 0.48 (Table 9). Similarly, posts made by group A were significantly more reliable compared to group B (p = 0.0001). The mean difference of reliability score was 0.44, with a 95% confidence interval ranging from 0.24 to 0.64 (Table 10).

**Table 9:** Unpaired t-test of global quality score.

	Group A (n = 200)	Group B (n = 231)
Mean ± standard deviation	2.42 ± 1.01	2.13 ± 1.02
Standard error	0.07	0.07
t	2.96	
Degree of freedom	429	
Standard error of difference	0.10	
Mean difference	0.29	
95% confidence interval	0.10–0.48	
p-value	0.0033	

**Table 10:** Unpaired t-test of reliability score.

	Group A (n = 200)	Group B (n = 231)
Mean ± standard deviation	2.34 ± 1.04	1.90 ± 1.03
Standard error	1.04	1.03
t	4.40	
Degree of freedom	429	
Standard error of difference	0.10	
Mean difference	0.44	
95% confidence interval	0.24–0.64	
p-value	0.0001	

## Discussion

Social media exposure has a positive correlation to seizure experiences. Recent research conducted by South et al. [14] stated that exposure to certain visual patterns on social media could trigger seizures among 3% of individuals with epilepsy. Particularly, videos and GIFs have severe effects on people with photosensitivity. Individuals with photosensitivity and reflex epilepsies should avoid much exposure to social media videos and GIFs that may trigger seizures [15]. According to social media posts from the United Kingdom, many people with seizures tend to post their experiences on Instagram to show others what it means to live with seizures. For instance, a 24-year-old model shared her experience with epileptic seizures on Instagram to “show the reality of living with epilepsy” [16]. Implicitly, social media is a critical platform for sharing information about seizures, yet its use may be positively related to increasing cases of epileptic seizures. As a platform to share information regarding seizures regardless of the background of the social media users (medical professionals, survivors of seizures, communities, organizations, and foundations), social media can also reshape a person’s understanding about certain information [17]. Therefore, when various individuals make Instagram posts about their experience with a seizure disorder, there is a need for the viewers to understand the quality and reliability of the post. It is essential in determining if the information in a post is true or false, thus helping in utilizing it appropriately. 

Our study analyzed the accuracy of information related to seizures on Instagram. Previous studies have analyzed seizure/epilepsy-related information on other social media platforms, such as Facebook, Twitter, and TikTok [8,9]. The results from the study indicate that the number of people engaging in Instagram posts concerning seizures was more than five hundred thousand likes and about four hundred comments. However, this number could be higher because most people do not engage with posts after viewing them. The health and wellness industry posted the most posts, followed by survivors, communities, organizations, advocates, and doctors. Many organizations in the wellness and health industry are shown to be actively engaging in creating awareness about seizures, their causes, treatment, and preventive approaches [18]. According to the results, the wellness and health industry is leading in posting about seizures on Instagram. This finding concurs with the recommendation made in the review by the Epilepsy Foundation that organizations in the health and wellness industry have spent countless efforts to combat seizures by actively engaging the public through social media [19]. 

In our study, most posts were about patient experiences (39.2%), symptomatology (37.8%), and treatment (20.0%). A previous study conducted by Jiang et al., which analyzed 109 epilepsy-related videos in TikTok, included personal experience videos (51%), event videos (47%), and educational videos (11%) [9]. Compared to a previous study by Meng et al., which analyzed epilepsy-related posts on Facebook and Twitter, 48% of them focused on epilepsy treatment and epilepsy-related misconceptions [8].

We divided the group types into group A and group B to know whether the information made by healthcare professionals and non-healthcare professionals would cause any differences in terms of correct information, quality, and reliability. Most of the Instagram posts contained true information regarding seizure and/or epilepsy (76.8%). Regarding the correct information in Instagram, group A (wellness and health industry, nurses, neuroscience physiotherapists, and dieticians) posted 168 correct information, while group B (patients, survivors, public figures, and other professionals) posted 163 correct information. This information indicates that there is a gap creating room for improvement, especially when it comes to creating awareness and sharing information about seizures. 

We further assessed the global quality score among posts made by groups A and B. The mean global quality score between groups A and B differed statistically (p = 0.0033). However, the mean difference was only 0.29, with a higher mean global quality score observed among group A (2.42 ± 1.01). Similarly, the mean reliability score between groups A and B differed statistically (p = 0.0001). However, the mean difference was only 0.44, with a higher mean reliability score observed among group A (2.34 ± 1.04). 

A total of 100 Instagram posts cannot be determined due to not mentioning the definition of epilepsy according to the WHO, CDC, and ILAE. We did not find any false information in Instagram posts. In contrast, a previous study by Jiang et al., which analyzed 109 epilepsy-related videos in TikTok, found misinterpretations of epileptic events in 26 out of 47 event videos [9].

Instagram information consumers must appreciate that the media reports are not foolproof, as they are made by different individuals who are not guided by a similar prompt. However, healthcare regulators (such as WHO) and healthcare professionals should work together to ensure that most of the information circulating on social media platforms originates from relevant sources, thus, enabling the information consumers to get quality information [17]. This way, if someone were checking Instagram posts on seizure disorders, they would be fed essential information in most cases instead of getting unreliable information. The interaction between the doctors and the health care regulators would enable the doctors to guard, feed Instagram users with professional posts about the disorders, and allow the social media users to benefit when understanding various concepts related to seizure disorders [8]. In the same way COVID-19, WHO popups should come up every time on Instagram telling users to verify the information they are consuming about seizure disorders through the WHO site, as this would facilitate accurate information, thus allowing them to interact with quality and reliable posts. Moreover, new tools, such as the utilization of machine learning algorithms, are needed to help assess the quality and accuracy of social media posts related to seizures.

Our study does have certain limitations. Firstly, we only looked at posts from one social media platform, i.e., Instagram, and no other platforms were used. Secondly, we did not look at all posts that popped up when using a certain hashtag. Another limitation can be related to calculating the engagement rate. The results captured only those who engaged with the posts through likes and comments. Thirdly, we analyzed the Instagram content from public Instagram accounts only. Thus, the result of this study did not represent the engagement of Instagram content from private Instagram accounts. 

## Conclusions

This current study provides a comprehensive reference on the usage of Instagram to assess the veracity of the information on seizure and/or epilepsy. Healthcare professionals and non-healthcare professionals may spread seizure-related information on Instagram. The users should be aware of the correct information, quality, and reliability of each seizure-related Instagram post. In the future, healthcare regulators and the utilization of machine learning can help to assess the quality and accuracy of social media posts related to seizures.
